# Identification of genes required for the survival of *B. fragilis* using massive parallel sequencing of a saturated transposon mutant library

**DOI:** 10.1186/1471-2164-15-429

**Published:** 2014-06-04

**Authors:** Yaligara Veeranagouda, Fasahath Husain, Elizabeth L Tenorio, Hannah M Wexler

**Affiliations:** GLAVAHCS, Bldg. 115 Room 312 11301 Wilshire Blvd, Los Angeles, CA 90073 USA; UCLA School of Medicine, Los Angeles, CA USA; Division of Geographic Medicine and Infectious Disease, Tufts Medical Center, Boston, MA 02111 USA

**Keywords:** *Bacteroides fragilis*, Transposon mutants, Essential genes, Massively parallel sequencing, COG, DEG

## Abstract

**Background:**

*Bacteroides fragilis* is a Gram-negative anaerobe that is normally a human gut commensal; it comprises a small percentage of the gut *Bacteroides* but is the most frequently isolated *Bacteroides* from human infections. Identification of the essential genes necessary for the survival of *B. fragilis* provides novel information which can be exploited for the treatment of bacterial infections.

**Results:**

Massive parallel sequencing of saturated transposon mutant libraries (two mutant pools of approximately 50,000 mutants each) was used to determine the essential genes for the growth of *B. fragilis* 638R on nutrient rich medium. Among the 4326 protein coding genes, 550 genes (12.7%) were found to be essential for the survival of *B. fragilis* 638R. Of the 550 essential genes, only 367 genes were assigned to a Cluster of Orthologous Genes, and about 290 genes had Kyoto Encyclopedia of Genes and Genomes orthologous members. Interestingly, genes with hypothetical functions accounted for 41.3% of essential genes (227 genes), indicating that the functions of a significant percentage of the genes used by *B. fragilis* 638R are still unknown. Global transcriptome analysis using RNA-Seq indicated that most of the essential genes (92%) are, in fact, transcribed in *B. fragilis* 638R including most of those coding for hypothetical proteins. Three hundred fifty of the 550 essential genes of *B. fragilis* 638R are present in Database of Essential Genes. 10.02 and 31% of those are genes included as essential genes for nine species (including Gram-positive pathogenic bacteria).

**Conclusions:**

The essential gene data described in this investigation provides a valuable resource to study gene function and pathways involved in *B. fragilis* survival. Thorough examination of the *B. fragilis*-specific essential genes and genes that are shared between divergent organisms opens new research avenues that will lead to enhanced understanding of survival strategies used by bacteria in different microniches and under different stress situations.

**Electronic supplementary material:**

The online version of this article (doi: 10.1186/1471-2164-15-429) contains supplementary material, which is available to authorized users.

## Background

The human gut is home to 10-100 trillion generally symbiotic bacteria that comprise the gut microbiome [1]. Some species become very pathogenic and cause serious infection if they escape their normal niche because of a compromised host gut (due to ulcers, cancer, trauma, surgery or other factors). *Bacteroides fragilis* is one such example. As a commensal it provides many benefits to the host, including digestion of complex polysaccharides, production of volatile fatty acids, bile acid recycling and immunity development. However, outside its niche *B. fragilis* can be an opportunistic pathogen [2–5].

While *B. fragilis* accounts for only a small percentage of the gut *Bacteroides*, it is the major *Bacteroides* species isolated from human infections [3]. While the scope of the factors that account for the particular virulence of *B. fragilis* are not fully known, several virulence factors have been described including the ability to withstand low concentrations of oxygen [6], release of degradative enzymes such as fibrinogenolysin [7], enterotoxin production, evasion of complement-mediated killing and phagocytosis, induction of abscess formation, and extensive within-strain variation of surface proteins and polysaccharides (PSs) [8, 9]. The capsular polysaccharides (CPS) that can induce abscess formation [10] have been extensively studied. Intra-abdominal abscesses can result in intestinal abstraction, erosion of resident blood vessels and ultimately fistula formation. Abscesses may also rupture and result in bacteremia [3, 4].

In many cases, treatment of *B. fragilis* infection is problematic owing to its high level of resistance to multiple classes of antibiotics. Many *B. fragilis* clinical isolates are resistant to aminoglycosides, β-lactams and macrolide antibiotics [11]. Resistance to metronidazole, the most widely prescribed antibiotic for *B. fragilis* infections, is also increasing [2, 11, 12]. Identification of essential genes (i.e., the genes that are indispensable for the survival of an organism under specific conditions) helps in defining targets for new antimicrobial agent development [13]. In addition, essential genes conserved across the species may serve as potential targets for designing broad-spectrum antibiotics. In fact, this approach was used in identifying new antimicrobial targets in *Burkholderia thailandensis*[13]. On the other hand, if a narrow spectrum was desired, an agent that targeted only functions specific to the pathogen could potentially be designed.

Recent advancement in sequencing technologies has allowed the simultaneous study of large mutant libraries and the subsequent identification of genes necessary for bacterial survival [14, 15] and has resulted in the identification of essential genes in many pathogenic bacteria such as *Mycobacterium tuberculosis*, *Salmonella typhimurium*, *Helicobacter pylori*, and *Pseudomonas aeruginosa*[16–19]. The results of many of these studies been collated in a Database of Essential Genes (DEG) [20].

We undertook this study to identify the essential genes of *B. fragilis* 638R. Essential gene identification has limitations irrespective of method used (either gene deletion or transposon gene disruption) [21]. One of the key factors for success in essential gene identification in bacteria is the generation of mutants. Mutants can be generated either using traditional methods such as gene deletion by homologous recombination or by using a transposon delivery vector. Although the traditional method may be more technically rigorous, it is very labor-intensive and therefore expensive [22, 23] especially in *B. fragilis* species that is less amenable for genetic manipulation due to its Restriction/Modification system [24]. On the other hand, transposon mutant generation is relatively easy but requires an efficient and unbiased transposon delivery vector. In addition, the advantage of transposon mutagenesis is that it allows the simultaneous study of the large number of mutants in a variety of conditions, thereby identifying the genes important or detrimental to growth in that particular condition. Interestingly, the mariner transposon vector pSAM_Bt, developed for use in *B. thetaiotaomicron*[14] was useful for constructing saturated transposon libraries of *B. fragilis* 638R, a frequently used strain in molecular studies of *B. fragilis*[25]. In addition, pSAM_Bt has been successfully used for essential gene identification in *B. thetaiotaomicron* and *P. gingivalis*[14, 21]. In the present investigation we generated a saturated mutant library using pSAM_Bt and identified the genes required for the survival of *B. fragilis*.

## Results and discussion

### Construction of transposon mutant library and mutants’ analysis

We previously described the technique of *B. fragilis* 638R transposon mutant library construction used in this analysis [25]. The pSAMBt mariner transposon that was designed for essential gene identification has 1) Illumina P7 sequencing adapters (P7) near inverted repeats that facilitate sequencing of mutants and 2) a two-hairpin motif downstream of the *ermF* cassette that prevents read-through of the transposon disrupted gene [14]. For the subsequent mutant analysis we used the procedure described in detail for the identification of the essential genes in *Porphyromonas gingivalis*[21].

We independently generated two ~50,000 mutant libraries (i.e., biological replicates MP1 and MP2). After the genomic DNA preparation, each of these samples was split into two technical replicates (TR) to minimize any changes due to technical variation introduced by downstream manipulations (i.e., shearing of mutant DNA, transposon junction recovery by C-tailing followed by PCR and NGS sequencing). These samples (MP1-TR1, MP1-TR2, MP2-TR1 and MP2-TR2) were used for the identification of transposon disrupted region as described [21].

**Figure 1:**
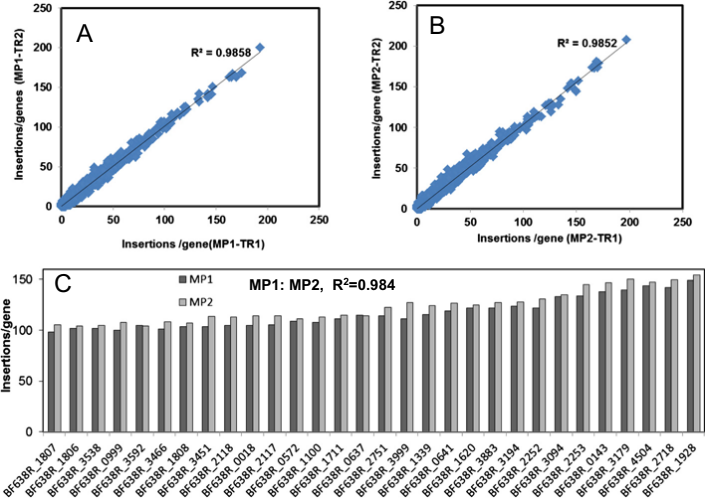
**Reproducibility of transposon mutant library.** The number of unique transposon insertions/gene between technical replicates of the mutant population 1 (MP1) **(A)** and 2 (MP2) **(B)**. Each point represents the number of unique insertions in the specific gene present in two technical replicates. **C**. Reproducibility between biological replicates. The number of unique insertions/gene in two technical replicates are averaged and then compared between biological replicates. Only genes with 100-147 unique insertions/gene are displayed. The R^2^ between MP1 and MP2 is 0.984.

Averages of 17 million reads were obtained for each sample in a multiplex run. After quality filtering and clipping, 13.5 ± 0.9 million reads per sample were mapped to the genome of *B. fragilis* 638R. The transposons inserted both within (93.5% reads) and between (6.5% reads) the genes. The number of unique insertion sites/gene between technical replicates showed good correlation; R^2^ values for technical replicates of MP1-TR1/MP1-TR2 and MP2-TR1/MP2-TR2 were 0.9858 and 0.9852, respectively (Figure 1A and B). We then averaged the number of unique insertion sites/gene of two technical replicates and compared the values of the biological replicates. The reproducibility between two biological replicates was also high; the number of unique insertion sites/gene in MP1 and MP2 gave an R^2^ value of 0.984. Figure 1C is a representation of those genes which had 100-147 unique insertions/gene in two biological replicates (we only included selected genes for figure clarity). The results confirm that mutant generation by the transposon vector and identification of the transposon disrupted genes is reproducible and reliable. Each of the biological replicates yielded mutant libraries of > 50,000 mutants (51,102 ± 779 mutants for MP1 and 59,001 ± 3251 mutants for MP2, respectively).

### Identification of candidate essential genes

*B. fragilis* 638R has 4326 protein coding genes, 72 tRNA genes and 19 rRNA genes [9]. We investigated the genes that were disrupted by transposons in two independent mutant pools. Genes that had transposon insertions in the last 5% of the gene (3' end) were filtered out since they may likely to generate active product and the remaining reads were mapped against the *B. fragilis* 638R genome [14]. Analysis of the mutant pool indicated that 3763 of the 4326 genes, 55 of the 72 tRNA genes and all nineteen rRNA genes were disrupted by the transposon. Of the 3763 disrupted genes, 201 were disrupted only once in either one or both the biological replicates. Closer examination of these 201 mutant reads indicated that transposon was integrated well within the genes. In addition, all 201 genes were disrupted in a mutant pool which was sequenced following re-growth of mutants on BHI medium, confirming that these genes are not essential for survival of *B. fragilis* 638R. There were 1764 genes with 1-5 disruptions and 1798 genes with 6-198 disruptions in both biological replicates. Thus, 3762 genes (~87%) can be individually disrupted without eliminating growth of *B. fragilis* 638R on BHI medium.

**Figure 2:**
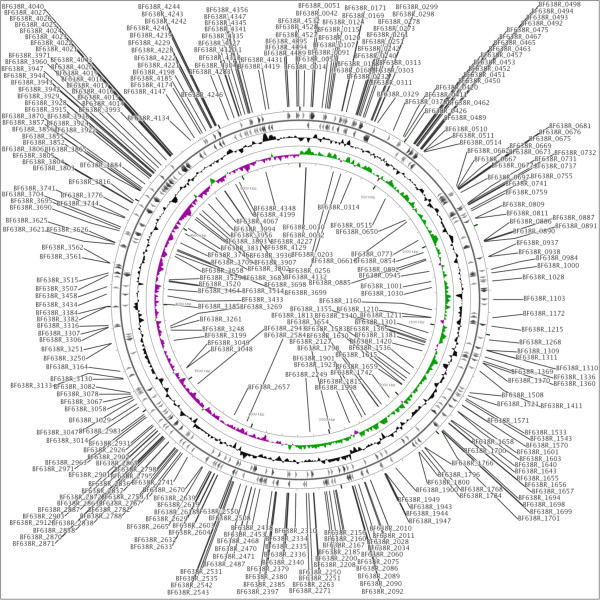
**Distribution of essential genes on** ***B. fragilis*** **638R genome.** Distribution of essential genes on positive (outside circle) or negative (inside circle) strands of *B. fragilis* 638R chromosome.

Genes were considered essential if they were not disrupted by the transposon in either biological replicate. Mariner transposons preferentially insert into TA sites, therefore, we disregarded genes which have either less than 10 TA sites or were less than 150 bp in length, since these genes are likely to escape random transposon disruption [14]. With these qualifications in place, 550 (12.7%) genes were classified as essential for growth of *B. fragilis* 638R on BHI medium. The essential genes were distributed evenly throughout the genome (Figure 2). The full list of essential genes along with KEGG ortholog numbers, KEGG pathways, COG classification is presented in Additional file 1: Table S1.

### COG and KEGG classification of essential genes

**Figure 3:**
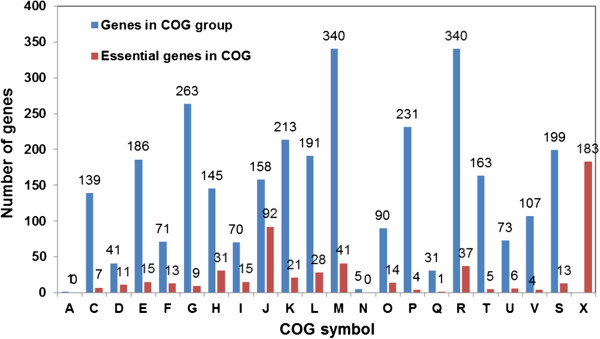
**COG Classification of** ***B. fragilis*** **638R essential genes.** Legend: A- RNA processing and modification, C-Energy production and conversion, D- Cell cycle control, cell division, chromosome partitioning, E- Amino acid transport and metabolism, F- Nucleotide transport and metabolism, G- Carbohydrate transport and metabolism, H- Coenzyme transport and metabolism, I- Lipid transport and metabolism, J- Translation, ribosomal structure and biogenesis, K- Transcription, L- Replication, recombination and repair, M- Cell wall/membrane/envelope biogenesis, N- Cell motility, O- Posttranslational modification, protein turnover, chaperones, P- Inorganic ion transport and metabolism, Q- Secondary metabolites biosynthesis, transport and catabolism, R- General function prediction only, T- Signal transduction mechanisms, U- Intracellular trafficking, secretion, and vesicular transport, V- Defense mechanisms, S- Function unknown, X- Essential genes not in COG.

We classified the essential genes according to the COG (Figure 3). Of the 550 essential genes, 367 (66.7%) genes are distributed in various domains of COGs and 290 genes belong to KEGG orthologous members (Additional file 1: Table S1). The majority of the essential genes code for proteins involved in basic cellular process such as translation, cell wall biogenesis, replication, recombination and repair, and transcription. The relative abundance of essential genes compared to total genes was highest in the COG group J (translation, ribosomal structure and biogenesis) (Figure 3 and Additional file 1: Table S1). Noticeably the essential gene list is missing (or has only a small representation) genes of COG groups for essential pathways suggesting that multiple genes can substitute for each other.

Four of the six subunits of DNA polymerase III holoenzyme (*dnaE*, BF638R_2865, BF638R_2439, BF638R_3948), DNA elongation and topology changing genes (*ligA*, g*yrA, gyrB* and *parE*), and a few, but not all, recombination repair genes (*ruvB*, *uvrD*, *ruvX*, *refC*, and *pol*A) were essential. In the transcription pathway, the core subunit of RNA polymerase (*rpoA*), transcription terminator (*rho*), antitermination protein (*nusG*), nitrogen utilization regulator (*nusA* and *nusB*) and several other sigma factors were essential. The translation, ribosomal structure and biogenesis COG group included many essential genes encoding 30S and 50S ribosomal proteins (BF638R_4015-BF638R_4045, and BF638R_4053- BF638R_4059) and all twenty aminoacyl tRNA synthetase genes were identified as essential genes. In addition, a few, but not all, genes involved in translation initiation (*infA*, *B* and *C*), elongation (*tsf*, *fusA* and *tuf*) and release factors (*frr*, *prfA* and *pth*) were essential.

The 41 essential genes in the cell wall/membrane/envelope biogenesis pathway code for proteins involved in peptidoglycan biosynthesis, LPS core region and lipid-A biosynthesis; proteins for *O*-antigen biosynthesis were not among the essential genes. In the signal transduction pathway, one two-component regulator was essential (*rprX*/*rprY*). The genes encoding for chaperones (heat shock protein) such as *groEL*, *groES*, *grpE* and *ftsH* (BF638R_0745) were also identified as essential for *B. fragilis* growth. Also, many genes involved in amino acid, nucleotide, lipid and cofactor metabolism were present in the essential gene list.

### Conjugation associated *tra* genes

Surprisingly, many conjugation related genes (such as *traB*, *traE*, *traF*, *traH*, *traF*, *traI*, *traQ*) were classified as essential genes. Similar conjugation transfer related genes were also present in the essential gene list of *B. thetaiotaomicron*[14] and particular domains of certain of the *tra* genes were not disrupted in *P. gingivalis*, although the other domains were disrupted [21]. The function of these genes outside of their importance in conjugation has not been described. The results suggest that they either have some function in cell viability or are somehow not available for transposon insertion.

### Capsular polysaccharides (CPS) biosynthesis, RND efflux pumps and many regulator genes are not essential genes

Interestingly the genes in the eight clusters involved in CPS biosynthesis, the sixteen RND efflux pump genes and the 32 *araC*-type regulator genes are not among the essential genes of *B. fragilis* 638R, although a few of them have been shown to be critical in *B. fragilis* under specific conditions. Since these gene classes are particularly redundant in *B. fragilis*, it is reasonable to presume that the deleted gene is complemented by homologous members. It would be tempting to speculate that the essential genes are more likely to be non-redundant with a critical function that is not complemented by homologous genes. It is of special interest that the two component transduction regulator, *rprX*, is essential in spite of having seventeen and thirty-six homologs respectively in the Database of Essential Genes (DEG) for *rprX* and *rprY*, respectively. The *B. fragilis rprX/rprY* genes expressed from a multicopy plasmid in *E. coli* affect the respective levels of the OmpF and OmpC porins, perhaps by interfering with normal regulation of OmpF [26] and in *P. gingivalis*, RprY appears to regulate stress responses [27].

### Essential genes of unknown function

Only 367 (66.7%) of the essential genes could be assigned to a COG functional category (Additional file 1: Table S1) and 44 of these genes had no specific function delineated. The remaining 183 (33.3%) essential genes that were not assigned to COG groups coded for hypothetical proteins. Thus, 227 (44 + 183) genes (41.3%) of the essential genes encode hypothetical proteins, demonstrating that the functions for nearly half of the genes critical for *B. fragilis* survival are still unknown.

### Comparison of *B. fragilis* 638R essential genes with related strains

**Figure 4:**
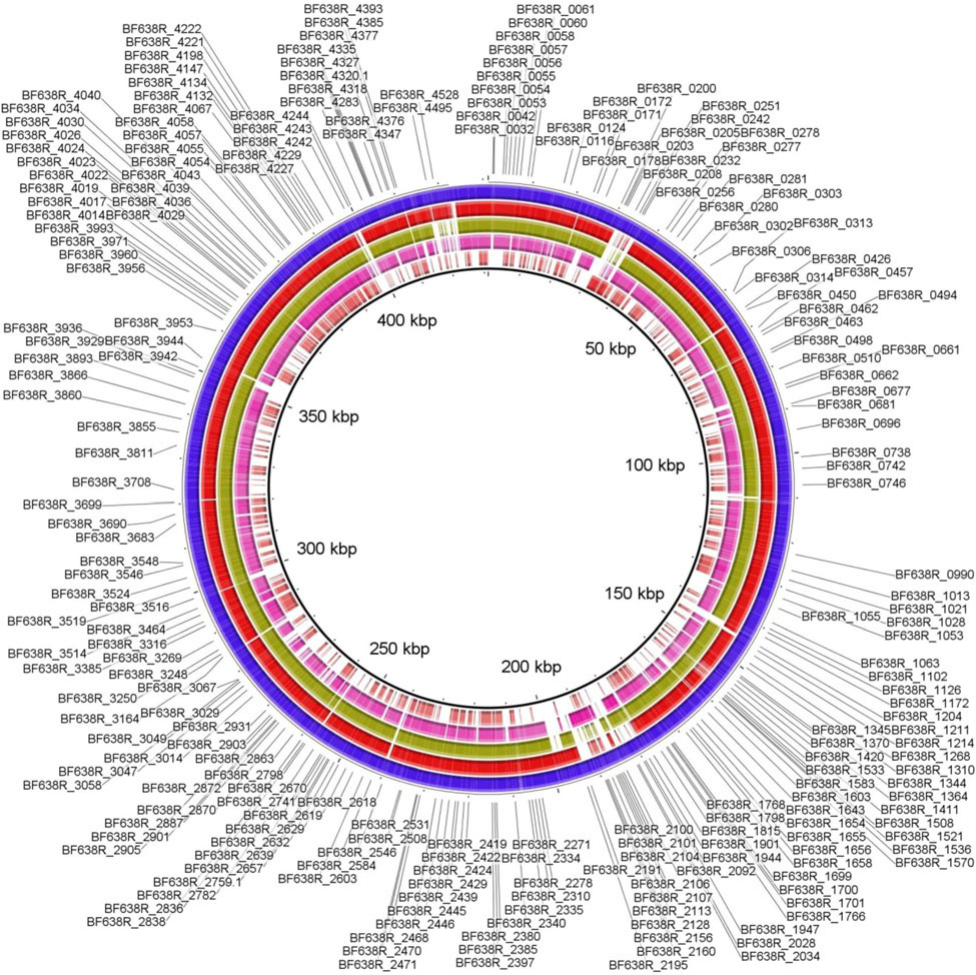
**Comparison of** ***B. fragilis*** **638R essential genes (blue circle) with** ***B. fragilis*** **9343 (red circle),** ***B. fragilis*** **YCH46 (green circle),** ***B. thetaiotaomicron*** **VPI-5482 (pink circle),** ***P. gingivalis*** **ATCC 33277 (light red).** Not all genes are labeled on the figure.

A whole genome comparison indicated that 88% (3812 and 3816 of 4326) of protein coding genes of *B. fragilis* 638R are conserved in *B. fragilis* 9343 and *B. fragilis* YCH46, respectively. Then we compared *B. fragilis* 638R essential genes with *B. fragilis* 9343 [24] and *B. fragilis* YCH46 [28]. Four hundred ninety four (90%) and 488 genes (89%) of the 550 essential genes of *B. fragilis* 638R were conserved in *B. fragilis* 9343 and *B. fragilis* YCH46, respectively (Figure 4 and Additional file 2: Table S2). Thirty-four essential genes in *B. fragilis* 638R (and annotated as hypothetical only) were missing from the other two *B. fragilis* strains, indicating that while the majority of the essential networks of *B. fragilis* 638R are likely conserved among the species but there are still differences between strains.

### Comparison of *B. fragilis* 638R essential genes with *B. thetaiotaomicron* VPI-5482 and *P. gingivalis* ATCC 33277

Genes needed in one bacterium are not necessarily essential in another species and comparing the essential genes of divergent species can provide valuable information about networks that are shared or not shared between organisms. We looked for homologs of *B. fragilis* essential genes in the related species *B. thetaiotaomicron* VPI-5482 (BT) and in the periodontal pathogen *P. gingivalis* ATCC 33277 (PG). The essential genes in BT and PG were previously identified using a saturated transposon mutant library [14, 21] and have 325 and 463 essential genes, respectively. Therefore, we also determined which of the essential genes of BF638R were also essential in those two species. Since BT and PG are less closely related to *B. fragilis* 638R, relatively fewer genes are conserved across the whole genome; sixty-nine percent (2986/4326) and 35% (1527/4326) of *B. fragilis* 638R genes are conserved in BT and PG respectively. Interestingly, 77% (425/550) and 64.6% (355/550) of the essential genes of *B. fragilis* 638R have close homologs in BT and PG*,* respectively (Figure 4, Additional file 2: Table S2) but not all of them are designated as essential in the other species. In fact, only 200 and 279 of the *B. fragilis 638R* essential genes are shared as essential genes by BT and PG, respectively (Additional file 2: Table S2). The majority of the conserved genes are highly enriched in certain COG groups (Additional file 2: Table S2). Many *B. fragilis* 638R essential genes (174, 47.41%) assigned to COG groups with critical functions (including chaperones (*grpE*, *groES*), recombination and repair (*polA*, *ruvB*, *ruvX*, *uvrD*), N-utilization regulator (BF638R_1213), thiamine biosynthesis (BF638R_2546, BF638R_2547) and many transcriptional regulators (BF638R_0733, BF638R_1336, BF638R_1533, BF638R_2028, BF638R_2310, BF638R_2798, BF638R_2903 and BF638R_3831) are, in fact, not essential for *B. thetaiotaomicron* VPI-5482.

Although *B. fragilis* and *P. gingivalis* live in widely different niches (gut and oral cavity), more *B. fragilis* essential genes are present in the oral anaerobe *P. gingivalis* ATCC 33277 essential genes than in the gut anaerobe *B. thetaiotaomicron* VPI-5482. The reasons for this are not clear. Essential genes with known function that are shared between *B. fragilis* 638R and *P. gingivalis* but not essential for *B. thetaiotaomicron* include 1) thirty-four genes belongs to translation, ribosomal structure and biogenesis, 2) eight genes involved in cell wall/membrane/envelope biogenesis, and 3) chaperones (BF638R_3251). In addition, all six genes involved in lipid-A biosynthesis are essential for *B. fragilis* 638R and *P. gingivalis* ATCC 33277 (*lpxA*, *lpxC, lpxD,* BF638R_0493, *lpxB*, and BF638R_3307), however only the latter three genes are essential in *B. thetaiotaomicron* VPI-5482 even though all of the six genes are present only in a single copy in the *B. thetaiotaomicron* VPI-5482 genome [14]. Thus, although *B. fragilis* 638R and *B. thetaiotaomicron* VPI-5482 are closely related species, they apparently rely on different sets of essential genes for their survival. Presumably, *B. thetaiotaomicron* has other homologs that code for these essential functions that were not picked up in the BLAST analysis for essential genes. About 21% (115) of the *B. fragilis* 638R essential genes which are missing in *B. thetaiotaomicron* and *P. gingivalis* are annotated as hypothetical proteins (Additional file 2: Table S2). Further study of the species and strain specific requirements of *B. fragilis* strains will help us to understand its abilities to adapt to specific microniches.

### Comparison of *B. fragilis* 638R essential genes with the database of essential genes (DEG)

**Figure 5:**
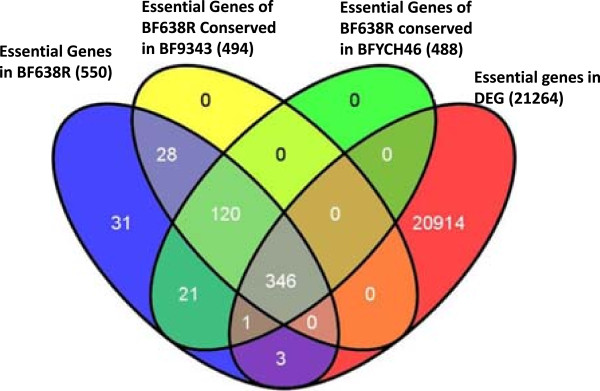
**Venn diagram of classifications of** ***B. fragilis*** **638R essential genes.** BF638R-*B. fragilis* 638R; BF9343-*B. fragilis* 9343; BFYCH46-*B. fragilis* YCH46; DEG-database of essential genes. There are 346 essential genes which are common to *B. fragilis* strains and have homologs in the DEG. Two hundred essential genes of *B. fragilis* 638R have no homologs in the DEG and 31 genes are specific to *B. fragilis* 638R.

We compared *B. fragilis* 638R essential genes with the Database of Essential Genes (DEG).10.02. The DEG Version 10.02 contains 21,264 essential genes and 646 essential non-coding sequences from 31 organisms [20]. *B. fragilis* 638R essential genes were compared with the genes listed in the DEG using their integrated BLAST function (E-value cutoff of <1.0 × 10^-5^) (Figure 5 and Additional file 3: Table S3).

The relationships between groups of essential genes between the *B. fragilis* strains and DEG is shown in a Venn diagram in Figure 5. Three hundred fifty (63.4%) of the 550 *B. fragilis* 638R essential genes are distributed in various bacterial species present in the DEG including Gram-positive bacteria such as *Bacillus subtilis* and *Staphylococcus aureus* (Table 1) [23, 29]*.* Thirty one percent of the essential genes of *B. fragilis* 638R have homologs in at least nine bacteria in the DEG (both pathogenic and non-pathogenic; and Gram-positive and negative), indicating that these genes may represent a core genome across bacterial genera (Table 1, Additional file 3: Table S3). Interestingly, 42 essential genes of *B. fragilis* 638R which are absent in both *B. thetaiotaomicron* VPI-5482 and *P. gingivalis* ATCC 33277 are conserved in other bacterial species in DEG. These 42 genes coded for proteins involved in vital functions such as arginine dependent acid resistance (BF638R_0188), chaperones (*grpE*, HSP70 co-factor), two-component sensor kinase (*rprX*), replication and repair functions (*polA*, *refC*, *ruvX*), translation machinery (9 genes), nucleotide metabolism and stringent response (BF638R_3808) (Additional file 3: Table S3). Why these 42 essential genes in *B. fragilis* 638R have homologs in phylogenetically diverse bacteria rather than BT or PG is not clear.

**Table 1:** **Comparison of essential genes of** ***Bacteroides fragilis*** **638R to other species in the DEG**

Bacteria	No. essential genes	Number of homologs of ***B. fragilis*** 638R essential genes present in other bacteria	% of ***B. fragilis*** 638R essential genes present in other bacteria
***B. fragilis*** **638R**	**550**	**-**	**-**
*P. gingivalis* ATCC 33277	463	277	50
*B. thetaiotaomicron* VPI-5482	325	211	36.5
*Caulobacter crescentus*	480	199	36
*Mycobacterium tuberculosis* H37Rv II	771	193	35
*Salmonella enterica serovar Typhi*	353	187	34
*Staphylococcus aureus* NCTC 8325	351	183	33
*Burkholderia thailandensis* E264	406	175	32
*Bacillus subtilis* 168	271	173	31
*E.coli* MG1655 II	296	171	31

**Figure 6:**
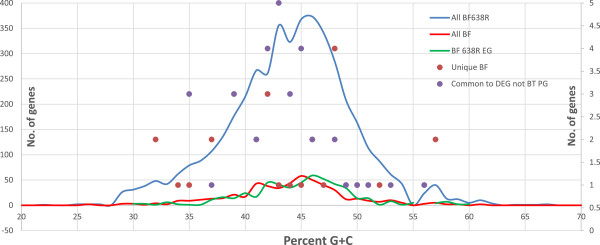
**Distribution of GC content in** ***B. fragilis*** **638R genes.** No. of genes with GC percentage indicated on Y-axis. Blue line: *B. fragilis* 638R genes. Green line: *B. fragilis* 638R essential genes. Red line: *B. fragilis* 638R essential genes that are common to other *B. fragilis species*. Red dots: *B. fragilis* 638R essential genes unique to *B. fragilis* 638R. Blue Dots: *B. fragilis* 638R essential genes with matches in the DEG but not to *P. gingivalis* or *B. thetaiotaomicron*. Note: The lines are mapped to the primary Y-axis and the dots to the secondary (right side) Y-axis.

The GC distribution of the total genes, essential genes and various groups of essential genes of *B. fragilis* 638R is shown in Figure 6. The GC% distribution of the essential gene set essentially matches that of the total genes, except for a few genes. Interestingly, many of the genes that were not found in *B. thetaiotaomicron* and *P. gingivalis* had GC% outside the normal distribution of *B. fragilis* 638R, suggesting that these genes may have been horizontally transferred from a phylogenetically diverse organism. Sixteen of these genes had GC% above 50 or below 40; thus it is possible that they recently transferred from another species and do not have close homologs in the related anaerobes (*B. thetaiotaomicron* or *P. gingivalis*)*.*

Most of the genes that had no homologs in the DEG (175/200) coded for a hypothetical protein. (This is expected because the annotation server depends on characterized homologs to assign homologs). The annotated protein sequences of these genes were also submitted to the Phyre2 server that predicts function based on conserved fold analysis; in some cases the Phyre2 server will predict function when the other servers do not. Phyre 2 analysis predicted functions for 4 genes which were previously annotated only as hypothetical proteins: BF638R_0208 and BF638R_0260 (PG016-like [2 helical bundles]), BF638R_2531 (hth-type transcriptional regulator) and BF638R_4199 (thioredoxin like) (Additional file 1: Table S1).

Transposon insertion in certain genes may not be tolerated, not because those genes themselves are essential, but because the disruptions may affect expression of downstream essential genes present within the same operon. Therefore, we analyzed potential polar effects of the *B. fragilis* 638R essential genes. Based on the operon prediction, disruptions in 74 of the 550 essential genes are likely to have a polar effect on downstream genes present in the same operon (Additional file 1: Table S1). These 74 genes are at the upstream end of an operon that contains one or more known essential genes as described in the DEG. Interestingly, 55 of those 74 genes are also present in the DEG; many of those genes are conserved in essential genes of more than one strain and deserve further study.

### Transcription analysis of essential genes

Whole genome transcription analysis by RNA-seq [30] indicated that 4093 of 4326 (94.6%) genes are transcribed in *B. fragilis* 638R. Transcription levels of essential genes demonstrated that most of the essential genes with assigned COG (320 of the 323) are transcribed (ribosomal genes, for example, are highly transcribed) (Additional file 1: Table S1). Also, 187/227 genes classified as hypothetical proteins are transcribed. Mid-log cells were used for RNASeq analysis, thus it is possible that the other 40 genes which did not show transcript/expression may be transcribed at a different growth stage. This data suggests that many genes with completely unknown function are essential for the growth of *B. fragilis* 638R.

## Conclusions

We identified the essential genes required for the survival of *B. fragilis* 638R in BHI medium using a transposon delivery vector and Illumina sequencing technology. The results indicate that only 12.7% (550) genes are essential. The *B. fragilis* genome is known for having redundant genes; for example, it has sixteen RND efflux pumps, at least four genes for the major membrane protein OmpA [31], more than 32 *araC*-type regulators [32], multiple operons for the degradation of dietary polysaccharides, and eight operons for capsular polysaccharides. In cases of gene redundancy, a disrupted gene may be complemented by another gene (presumably a homolog), and it would be expected that genes with multiple homologs might not be essential for survival of *B. fragilis* 638R but will result in synthetic lethality if all homologs are targeted. Thus, most of the essential genes described for *B. fragilis* 638R are genes which do not have a complementary gene. Further study is needed to determine why specific genes are essential, particularly for the 227 genes code for hypothetical proteins with no known function. Since most of the essential genes code for proteins that are involved in fundamental biological process such as translation, cell wall biogenesis, translation and transcription, we presume that 277 hypothetical genes also encode for proteins in vital pathways. Further characterization of these hypothetical proteins may provide novel information about unique pathways used by *B. fragilis*.

## Methods

### Strains and culture conditions

The *B. fragilis* 638R used in this study was originally isolated from an abdominal abscess [9]. *B. fragilis* and *E. coli* were grown in brain heart infusion (BHI) and LB broths, respectively, at 37°C. *E. coli* Top10 (Invitrogen, NY, USA) *and E. coli* S-17-1 λ pir strains were used as the host for cloning. *E. coli* S17-1 λ *pir* contains the *pir* gene and has chromosomally integrated conjugational transfer functions (RP4/RK6) so that bi-parental mating can take place in lieu of tri-parental mating using helper strains.

### Transposon mutagenesis and mutant library construction

*B. fragilis* was mutagenized using the mariner transposon vector as described previously [25]. Fifteen independent mating mixes (1 ml each) were pooled (15 ml) and stored as 1 ml aliquots at -80°C. Frozen aliquots were thawed and plated on BHI/gentamycin (25 μg/ml)/erythromycin (10 μg/ml)/rifampin (10 μg/ml) plate and incubated at 37°C for 3 days. The two mutants pools were generated by scraping the growth (approximately 50,000 mutant colonies) from the plate and suspending in 15 ml of LB/glycerol (20% v/v) medium. The resultant fifty thousand mutant pools were stored as 1 ml aliquots at -80°C.

### Sequencing mutants and mapping mutated genes

Four tubes of stored glycerol stocks of the mutant pool were used to make genomic DNA. The genomic DNA was prepared using ZR Fungal/Bacterial DNA MidiPrep™ kit (Zymo Research Corporation, CA). The technical replicates for each mutant pool were prepared by splitting genomic DNA into two. The transposon mutants in the mutant pool were identified essentially as described by Klein *et al*. [21].

### DNA shearing and adding C-tail

The genomic DNA from the mutants (10 μg) was sheared to 300 to 500 bp at the Biomedical Genomics Core Facility (San Diego) using the Covaris E220 focused ultrasonicator. C-tails were added to the sheared DNA using the terminal transferase kit (New England Biolabs, MA) and the chain terminator ddCTP (GE Healthcare Biosciences, NJ). C-tailing was carried out in a 60 μl reaction mixture (6 μl of 10X buffer, 6 μl of 2.5 mM CoCl2, 3 μl of dCTP (9.5 mM) –ddCTP (0.5 mM) mix, 3 μg of sheared DNA and 3 μl terminal transferase (20 units/μl) with the appropriate volume of water. The reaction mix was incubated at 37°C for 60 minutes and heat inactivated by incubating at 70°C for 10 minutes. The reaction mixture was purified using DNA Clean & Concentrator™ (Zymo Research Corporation, CA) and eluted with 15 μl elution buffer.

### Transposon junction amplification, adding Illumina adaptors and indexing sites

Sheared/C-tailed DNA was amplified with primers that would amplify fragments containing the transposon IRR (inverted repeat right) along with the mutant junction DNA; the amplification was carried out in a 150 μl reaction mixture containing 15 μl C-tailed DNA as template, 75 μl Phusion® High-Fidelity PCR Master Mix (New England Biolabs, MA), 3 μl of 30 μM- olj376 (5' GTGACTGGAGTTCAGACGTGTGCTCTTCCGATCTGGGGGGGGGGGGGGGG 3'), 3 μl 30 μM- pSAM1 (5' CCTGACGGATGGCCTTTTTGCGTTTCTACC 3') primers and the appropriate volume of water. The 150 μl reaction mixture were split into 3 tubes (50 μl each) and the PCR conditions were: 2 min at 95°C, 24 cycles of 10 s at 95°C, 30 s at 60°C, and 60 s at 72°C followed by a final extension for 1 min at 72°C. All three reactions were pooled and used as template to add Illumina sequencing and indexing sites. The second PCR consisted of 4 μl of first PCR product as template, 100 μl Phusion® High-Fidelity PCR Master Mix, 88 μl water, 4 μl of 30 μM pSAM2 (5' AATGATACGGCGACCACCGAGATCTACACTCTTTGACCGGGGACTTATCATCCAACCTGTTA 3') and 4 μl of 30 μM indexing primer (5' CAAGCAGAAGACGGCATACGAGATNNNNNNGTGACTGGAGTTCAGACGTGTGCTCTTCCGATCT 3'). The 200 μl reaction mixture was split into 4 tubes (50 μl each); PCR conditions were 2 min at 95°C, fourteen cycles of 10 s at 95°C, 30 s at 52°C, and 120 s at 72°C followed by a final extension for 5 min at 72°C. The samples were then pooled and purified using the QIAquick PCR Purification Kit (QIAGEN, Valencia, CA) and eluted with 30 μl elution buffer.

### Sequencing mutant junctions and mapping to the genome

The amplified DNA fragments were sequenced on a single end Illumina flow cell using the Genome Analyzer II (TUCF Genomics, MA), for 51 cycles with custom primer which binds to IRR (pSAM3 -5' ACACTCTTTGACCGGGGACTTATCATCCAACCTGTTA 3') of the transposon DNA and the standard Illumina index sequencing primer. Generated FASTQ files were analyzed essentially as described [21] at Tufts University Galaxy server (http://galaxy.med.tufts.edu/) using *B. fragilis* 638R as the reference genome. Mapped reads are normalized as number of unique insertions per gene and compared between mutant libraries.

### Expression analysis

Mid-log cells of *B. fragilis* grown on BHI broth were harvested and RNA was prepared using the RNeasy minikit with RNAprotect bacterial reagent (QIAGEN, Valencia, CA). Purified total RNA was again treated with RNase-free DNase kit (QIAGEN, Valencia, CA). Following RNase-free DNase treatment, reduction in genomic DNA in the RNA sample was confirmed by qRTPCR; RNase-free DNase treatment effectively reduced genomic DNA contamination by >1000 fold. The majority of the rRNA (>95%) was removed from total RNA using the MICROBExpress™ Bacterial mRNA Enrichment Kit (Life Technologies Corporation) leaving enriched RNA. The cDNA was prepared from enriched mRNA using the SuperScript® Double-Stranded cDNA Synthesis Kit (Invitrogen™) and subjected to RNA-Seq at Otogenetics (Norcross, USA). The RNA-Seq files were analyzed using the Lasergene Genomics Suite (DNASTAR, Inc, Madison, USA).

### Bioinformatic analysis

The GenBank files (.gbk) of specific bacteria were downloaded from the National Center for Biotechnology Information ftp server. (ftp://ftp.ncbi.nih.gov/genomes/Bacteria/). The cluster of orthologous genes (COG) classification of *B. fragilis* as well as genome comparisons were from the Integrated Microbial Genomes (IMG) database (https://img.jgi.doe.gov/cgi-bin/w/main.cgi) [33]. The circular maps were constructed using the BLAST Ring Image Generator (BRIG0.95) (http://sourceforge.net/projects/brig/) [34] or the CGView Server (http://stothard.afns.ualberta.ca/cgview_server/). The formats of sequence file were converted as needed for subsequent analysis at http://sequenceconversion.bugaco.com/converter/biology/sequences/. *B. fragilis* essential genes were compared with the essential genes of other bacteria at the Database of Essential Genes (DEG).10.02 (http://tubic.tju.edu.cn/deg/) [20]. The distribution of *B. fragilis* essential genes in various pathways was investigated using the Omics Viewer with *B. fragilis* 638R as reference genome at http://biocyc.org/overviewsWeb/celOv.shtml and the Kyoto Encyclopedia of Genes and Genomes (KEGG). The KEGG entry number for *B. fragilis* 638R is T01691. The KEGG orthologous genes and KEGG pathways for *B. fragilis* 638R essential genes were obtained from the KEGG database (http://www.genome.jp/dbget-bin/www_bget?gn:T01691). We also used PHYRE2 analysis to predict protein function based on fold recognition patterns [35].

## Electronic supplementary material

Additional file 1: Table S1: Essential genes of *Bacteroides fragilis* 638R. (XLSX 62 KB)

Additional file 2: Table S2: Comparison of *B. fragilis* 638R essential genes with related strains and essential genes of *B. thetaiotaomicron* VPI-5482 and *P. gingivalis* ATCC 33277. (XLSX 60 KB)

Additional file 3: Table S3: Comparison of *B. fragilis* 638R essential genes with other strains in DEG. (XLSX 102 KB)
